# SRSF10 inhibits biogenesis of circ-ATXN1 to regulate glioma angiogenesis via miR-526b-3p/MMP2 pathway

**DOI:** 10.1186/s13046-020-01625-8

**Published:** 2020-06-29

**Authors:** Xiaobai Liu, Shuyuan Shen, Lu Zhu, Rui Su, Jian Zheng, Xuelei Ruan, Lianqi Shao, Di Wang, Chunqing Yang, Yunhui Liu

**Affiliations:** 1 Department of Neurosurgery, Shengjing Hospital of China Medical University, Shenyang, 110004 China; 2 Liaoning Clinical Medical Research Center in Nervous System Disease, Shenyang, 110004 China; 3 Key Laboratory of Neuro-Oncology in Liaoning Province, Shenyang, 110004 China; 4 Department of Neurobiology, School of life Sciences, China Medical University, Shenyang, 110122 China; 5 Key Laboratory of Cell Biology, Ministry of Public Health of China, China Medical University, Shenyang, 110122 China; 6 Key Laboratory of Medical Cell Biology, Ministry of Education of China, China Medical University, Shenyang, 110122 China

**Keywords:** Glioma, SRSF10, Circ-ATXN1, miR-526b-3p, Glioma associated endothelial cells, Angiogenesis

## Abstract

**Background:**

Angiogenesis plays an important role in the progress of glioma. RNA-binding proteins (RBPs) and circular RNAs (circRNAs), dysregulated in various tumors, have been verified to mediate diverse biological behaviors including angiogenesis.

**Methods:**

Quantitative real-time PCR (qRT-PCR) and western blot were performed to detect the expression of SRSF10, circ-ATXN1, miR-526b-3p, and MMP2/VEGFA. The potential function of SRSF10/circ-ATXN1/miR-526b-3p axis in glioma-associated endothelial cells (GECs) angiogenesis was further studied.

**Results:**

SRSF10 and circ-ATXN1 were significantly upregulated in GECs compared with astrocyte-associated endothelial cells (AECs). Knockdown of SRSF10 or circ-ATXN1 significantly inhibited cell viability, migration and tube formation of GECs where knockdown of SRSF10 exerted its function by inhibiting the formation of circ-ATXN1. Moreover, the combined knockdown of SRSF10 and circ-ATXN1 significantly enhanced the inhibitory effects on cell viability, migration and tube formation of GECs, compared with knockdown of SRSF10 and circ-ATXN1, respectively. MiR-526b-3p was downregulated in GECs. Circ-ATXN1 functionally targeted miR-526b-3p in an RNA-induced silencing complex. Up-regulation of miR-526b-3p inhibited cell viability, migration and tube formation of GECs. Furthermore, miR-526b-3p affected the angiogenesis of GECs via negatively regulating the expression of MMP2/VEGFA.

**Conclusion:**

SRSF10/circ-ATXN1/miR-526b-3p axis played a crucial role in regulating the angiogenesis of GECs. The above findings provided new targets for anti-angiogenic therapy in glioma.

## Background

Glioma is one of the most common primary intracranial tumors. Its resistance to chemoradiation leads to high recurrence rates after surgery and poor prognosis [1]. As a solid tumor, the poor survival of glioma is caused by its abundant angiogenesis, strong invasiveness, and therapeutic resistance [2, 3]. Excessive angiogenesis has become an indicator for glioma growth [4]. Angiogenesis is the process where new vessels arise from existing vessels, participating in a variety of physiological and pathological process. Abnormality in vessel proliferation is necessary for the growth, proliferation and migration of most solid tumors [5]. Therefore, investigation into the mechanism of glioma angiogenesis may provide new insights and reveal molecular targets for therapeutic research of glioma.

RNA binding proteins (RBPs) facilitate various physiological and pathological regulation in cells, including RNA splicing, modification, transport, localization, stability, degradation, and translation [6]. RBPs combine with target RNAs to form ribonucleoproteins (RNPs), exerting various effects in post-transcriptional regulation of genes [7]. Studies have shown that the interaction between RBPs and circRNAs affects the latter’s formation, transcription, post-transcriptional regulation, translation and extracellular transport [8–11]. SRSF10 (serine and arginine rich splicing factor 10), a member of SR protein family, acts as a sequence-dependent splicing regulator. It has been shown to play a role in muscle development, myoblast differentiation, and glucose production [12]. SRSF10 is also a critical factor in the regulation of adipocyte differentiation via controlling the production of Lipin1α [13]. Overexpression of SRSF10 promotes the development of colon cancer via regulating BCLAF1 splicing [14]. The function of SRSF10 in glioma angiogenesis is still unclear.

Circular RNAs (circRNAs), a kind of non-coding RNAs, are widely expressed in eukaryotes which have a covalently closed loop and no poly-adenylated tail [15]. Recent studies have revealed that circRNAs, binding with miRNAs as their sponges, regulate the splicing and translation of target genes [16]. CircRNAs can also exert biological functions by interacting with RBPs [17]. ATXN1 (ataxin 1) is involved in the development of neurodegenerative diseases [18, 19]. Circ-ATXN1 (circbase ID:hsa-circ-0075686) is located on chr6: 16299342–16,328,701. Its splicing product circ-ATXN1 is 9825 bp in length and consists of 2 exons. In the present study, we have verified that circ-ATXN1 is overexpressed in glioma-associated endothelial cells (GECs). Thus, we presume that circ-ATXN1 participates in the regulation of glioma angiogenesis and the specific mechanism of circ-ATXN1 function needs further exploration.

MiRNAs are non-coding RNAs with a length of 18-23 nt. MiRNAs bind to 3’UTR region of target genes to suppress translation or facilitate degradation of their mRNAs [20]. The regulatory effects of miRNA on cellular biological functions have become a research hotspot. Melatonin-mediated miR-526-3p upregulation in bone marrow-derived mesenchymal stem cells (BMSCs) promotes phosphorylation of SMAD1 to facilitate chondrogenic differentiation of BMSCs [21]. MiR-526-3p is lowly expressed in colon cancer cells. Upregulation of miR-526-3p modulates proliferation, invasion and glycolysis of colon cancer cells via inhibiting HIF-1α [22]. No research about circ-ATXN1 and miR-526-3p on glioma angiogenesis has been conducted.

MMP2 (Matrix metalloproteinase-2), a member of MMP family, is a zinc-dependent enzyme capable of cleaving components of the extracellular matrix and regulating signal transduction. MMP2 is critical in angiogenesis of nervous system and is also involved in growth, invasion and migration of tumors [23, 24]. VEGFA (Vascular endothelial growth factor-A) is a primary factor driving expansion of the tumor vascular bed [25]. VEGFA is involved in the regulation of various tumor angiogenesis (such as lung squamous cell carcinoma [26], ovarian cancer [27], etc.). MMP2 and VEGFA are both recognized as the molecular biomarkers of tumor angiogenesis.

Our study first verified the endogenous expression of SRSF10, circ-ATXN1, and miR-526b-3p. In addition, we explored their effects and interactions on angiogenesis in GECs. We further investigated SRSF10/ circ-ATXN1/ miR-526b-3p axis on glioma angiogenesis, in order to provide new theoretical and experimental basis in regulating glioma angiogenesis. In this study, our findings may provide a potential novel molecular therapy network for glioma anti-angiogenesis therapy.

## Materials and methods

### Clinical specimens

All human normal brain tissues and glioma specimens were obtained from the Department of Neurosurgery of Shengjing Hospital, China Medical University. All participants signed and provided informed consent. In the present study, the research methods were approved by the Institutional Review Board at Shengjing Hospital. The glioma tissues were classified by two experienced clinical pathologists into two groups, that is, LGGs (low grade gliomas: WHO grade I-II) and HGGs (high grade gliomas: WHO grade III-IV), according to the WHO classification.

### Cells lines and culture

The immortalized human cerebral microvascular endothelial cell line hCMEC/D3 was provided by Dr. Couraud (Institut Cochin, Paris, France). Cells applied in this study were within 28–32 passages. Endothelial cells (ECs) were cultured in endothelial basal medium (EBM-2; Lonza, Walkersville, MD, USA). Human glioma U87MG and human embryonic kidney 293 T (HEK293T) cell lines were obtained from the Shanghai Institutes and they were cultured in Dulbecco’s modified Eagle’s medium (DMEM) of high glucose with 10% fetal bovine serum. Primary NHA were acquired from the Sciencell Research Laboratories (Carlsbad, CA) and cultured under the conditions instructed by the manufacturer. All cells were maintained in a humidified incubator at 37 °C with 5% CO_2_. Glioma conditioned medium and astrocyte conditioned medium were collected as previously described [28]. Astrocyte conditioned medium was used as a negative control (NC).

### Cell viability assay

Cell Counting kit-8 (CCK-8) assay (Beyotime Institute of Biotechnology, Jiangsu, China) was performed to determine the viability of GECs. Cells were seeded in 96-well plates in triplicate and incubated in glioma conditioned medium for 24 h, respectively. Each well was incubated with 10 μl CCK-8 for 2 h and the absorbance was measured at 450 nm using a spectrophotometer (Molecular Devices, United States).

### Cell migration assay

GECs migration in vitro was assessed using a Transwell chamber (Costar, Corning, NY, USA) with a polycarbonic membrane (6.5 mm in diameter and 8 μm pore size). GECs were suspended into single cells in serum-free medium at the density of 5 × 10^5^ cells/ml. 100 μl suspension was added to the upper compartment and 600 μl of glioma conditioned medium supplemented with 10% FBS was added into the lower chamber. Cells were incubated for 48 h at 37 °C. Non-migrating cells on the top surface of membrane were removed with cotton swabs. Cells that migrated to the lower surface of the membrane were fixed with 3:1 methanol: glacial acetic acid, stained with 10% Giemsa solution for 30 min at 37 °C, and washed twice with phosphate buffer saline (PBS). Then the pictures of stained cells were taken with an inverted microscope. Then, stained cells in five randomly fields were randomly chosen for statistics.

### Fluorescence in situ hybridization (FISH)

To identified circ-ATXN1 and miR-526b-3p rearrangement in GECs, circ-ATXN1 probe (green-labeled; Biosense, Guangzhou, China) and miR-536b-3p probe (red-labeled; Exiqon, Copenhagen, Denmark) were used. Briefly, Slides were blocked with prehybridization buffer (3% BSA in 4× saline-sodium citrate, SSC). Then, the slides were treated with PCR-grade proteinase-k (Roche Diagnostics, Mannheim, Germany). The hybridization mix was prepared with circ-ATXN1 probe or miR-536b-3p probe in hybridization solution. And the slides were washed with washing buffer. Then the sections were stained with anti-digoxin rhodamine conjugate (1:100, Exon Biotech Inc., Guangzhou, China) at 37 °C for 1 h. Subsequently, the sections were stained with 4′,6-diamidino-2-phenylindole (DAPI, Beyotime, China). All fluorescence images (100×) were captured using a fluorescence microscope (Leica, Germany).

### Microarrays assay

RNA binding protein, circ-RNA, and miRNA analysis, Sample preparation and microarray hybridization were performed by Kangchen Bio-tech (Shanghai P.R. China).

### Reporter vector construction and luciferase reporter assay

HEK293T cells were seeded into a 96-well plate. The potential binding sequence of miR-526-3p in circ-ATXN1 gene and its mutant sequence was amplified by PCR, synthesized and cloned into the pmirGLO dual-luciferase vector (Promega, Madison, WI, USA). Wild-type pmirGLO-circ-ATXN1 (or circ-ATXN1 mutant) reporter plasmid and miR-526-3p agomir (or agomir NC) were co-transfected into HEK293T cells, when they reached 50–70% confluence. The pmirGLO empty vector was transfected as “Control” group. Luciferase activity was measured at 48 h after transfection through the Dual-Luciferase Reporter System (Promega). Wild-type MMP2–3′UTR reporter plasmid (MMP2-wt) and mutated-type MMP2–3′UTR reporter plasmid (MMP2-mut) were constructed with pmirGLO-promoter vector. Following transfection approach and measurement of luciferase activities were performed as described above.

### RNA-binding protein immunoprecipitation (RIP) assay

RIP was performed using a Magna RNA-binding protein immunoprecipitation kit (Millipore, Billerica, MA, USA) following the manufacturer’s instructions. Bio-pre-circ-ATXN1-Wt1 (5′ flanking, 5′-500 nt), Bio-pre-circ-ATXN1-Wt2 (3′ flanking, 3′ + 500 nt), and Bio-pre-circ-ATXN1-Wt3 (5′-500 nt + 3′ + 500 nt) were constructed for possible SRSF10 binding sites. In addition, Bio-pre-circ-ATXN1-Mut1 (5′ flanking, 5′-500 nt), Bio-pre-circ-ATXN1-Mut2 (3′ flanking, 3′ + 500 nt), and Bio-pre-circ-ATXN1-Mut3 (5′-500 nt + 3′ + 500 nt) for possible SRSF10 binding sites were constructed respectively. IgG (Millipore) was used as a control. Three wild-type circATXN1 or three mutant circATXN1 of SRSF10 binding site plasmids were transfected into GECs respectively. The Cells were lysed in complete RNA lysis buffer containing magnetic beads conjugated with human anti-SRSF10 antibody. The samples were incubated with Proteinase K and then immunoprecipitated RNA was isolated. Purified RNA was obtained and then applied in qPCR with reverse transcription analysis.

### Plasmid construction and cell transfection

Silencing plasmid of circ-ATXN1 was constructed in pGPU6/Hygro vector (Genechem Co, Shanghai, China). And a non-targeting sequence was used as a NC. Silencing plasmids of SRSF10 (NM_054016.3) with pGPU6/GFP/Neo (GenePharma, Shanghai, China) was constructed. Overexpression plasmid of SRSF10 and circ-ATXN1 were constructed in pIRES/EGFP vector (GenScript, Piscataway, NJ, USA). An empty vector was used as a blank control. GECs were seeded in 24-well plates and transfected using Opti-MEM I and LTX and Plus reagents (Life Technologies) when they were at about 80% confluence. Geneticin (G418; Sigma-Aldrich, StLouis, MO, USA) and Hygromycin (Solarbio, China) were selected. Furthermore, miR-526b-3p agomir (miR-526-3p (+)), miR-526-3p antagomir (miR-526-3p (−)), and their NC sequence (miR-526-3p (+) NC and miR-526-3p (−)NC) were synthesized (GenePharma, Shanghai, China) and transiently transfected into GECs using Opti-MEM and Lipofectamine 3000 reagents (Life Technologies Corporation, Carlsbad, CA, USA), respectively. Cells were collected 48 h after transfection. Sequences of sh-circ-AXTN1, sh-SRSF10 and sh-NC were shown in Table 1. The transfection efficiency of SRSF10, circ-AXTN1, and miR-526b-3p were shown in Supplementary Figure S2A-C.

**Table 1:** Sequences of shRNA template

Gene		Sequence(5’->3’)
SRSF10	Sence	GATCCgctgaagacgctttacataatTTCAAGAGA
		attatgtaaagcgtcttcagcTTTTTTGCTAGCG
	Antisence	AATTCGCTAGCAAAAAAgctgaagacgctttacataat
		TCTCTTGAAattatgtaaagcgtcttcagcG
NC	Sence	CACCGTTCTCCGAACGTGTCACGTCAAGA
		GATTACGTGACACGTTCGGAGAATTTTTTG
	Antisence	GATCCAAAAAATTCTCCGAACGTGTCACGT
		AATCTCTTGACGTGACACGTTCGGAGAAC
Circ-ATXN1	Sence	CACCAACAGTGCCAATTGCACATCCTCAAG
		AGGGATGTGCAATTGGCACTGTTTTTTTTG
	Antisence	GATCCAAAAAAAACAGTGCCAATTGCACTC
		CCTCTTGAGGATGTGCAATTGGCACTGTT

### Gelatin zymography

The gelatinolytic activity was identified by gelatin zymographic analysis, as previously described [29]. In brief, the total of 10 ml protein from the GECs was subjected to electrophoresis. Then, the gels were washed twice using 2.5% Triton X-100 in 50 mM Tris-HCL for 30 min and were incubated in calcium assay buffer overnight at 37 °C. The gels were stained with Coomassie Brilliant Blue in 50% methanol and 10% acetic acid and destained in 10%acetic acid. Finally, the gels were scanned by densitometry using CS Analyzer 3.0.

### Quantitative real-time PCR (qRT-PCR)

Total RNA was extracted from the cultured cells with Trizol reagent as described by the manufacturer (Life Technologies Corporation, Carlsbad, CA, USA). One Step PrimeScript™ RT-PCR Kits (Takara, RR064A, Japan) were used for measurement of circ-ATXN1. In addition, RNase-R was used to confirm the existence of circ-ATXN1 and eliminate the influence of liner RNAs. The expression of miR-526b-3p and U6 were detected via TaqMan MicroRNA Reverse Transcription kit and Taqman Universal Master Mix II (Applied Biosystems). One Step SYBR® PrimeScript™ RT-PCR Kit (Takara Biomedical Technology, Dalian, China) was used for the detections of SRSF10 and GAPDH. Their expressions were normalized to endogenous control GAPDH and fold change was determined as 2^−ΔΔCt^ in gene expression. Primers and probes used in this study are shown in Table 2.

**Table 2:** Primer and probes used for RT-qPCR

Primer or Probe	Gene	Sequence(5’->3’) or Assay ID
Primer	SRSF10	F:CCAGGGGGATCGAAAGACAC
		R:GTGGTCTTCCAGTCGGTCTAC
	Circ-ATXN1	F:GTGCTTGCAGGTTTTCTAGGTAG
		R:GTGGGTACAATCCGCCAAC
		P:FAM+AGCTCTGGATGTGCAATTGGCACTG+BHQ1
	GAPDH	F:GGACCTGACCTGCCGTCTAG
		R:TAGCCCAGGATGCCCTTGAG
		P:FAM+CCTCCGACGCCTGCTTCACCACCT+Eclipse
Probe	miR-526-3p	002383 (Applied biosystems)
	U6	001973 (Applied biosystems)

### Tube formation assay

Matrigel assay was performed to evaluate in vitro angiogenesis activity via quantifying tube formation as previously described. In total, 96-well culture plates were coated with 100 μL Matrigel (BD Biosciences, Bedford, MA, USA) per well and then allowed to polymerize for 30 min at 37 °C. Then, cells were resuspended in 100 μl glioma conditioned medium at a density of 4 × 10^5^cells/mL and added to Matrigel-coated wells. After maintained in 37 °C for 6 h, digital camera system (Olympus, Tokyo, Japan) was used to acquire three or more images at random from each culture, and Image J software was used to measure the number of tubes.

### Western blot assay

Total proteins from the cells were lysed with ice-cold RIPA buffer with protease inhibitors (Beyotime Institute of Biotechnology). Equal amounts of each protein samples (40 μg) was run on SDS/PAGE gels, transferred to PVDF membranes. Membranes were incubated in 5% fat-free milk in TBST and then incubated with primary antibodies against SRSF10 (1:500, Proteintech, Chicago, IL, USA), MMP2 (1:500, Proteintech, Chicago, IL, USA) and GAPDH (1:1000, Proteintech, Chicago, IL, United States).

### In vivo Matrigel plug assay

To measure the angiogenesis, Matrigel plug assay was conformed as previously described [30]. We purchased nude mice from the National Laboratory Animal Center (Beijing, China). Four-week-old BALB/c athymic nude mice were fed with autoclaved water and food during the experiment. All the experiments with the nude mice were performed strictly in according to the protocol approved by the Administrative Panel on Laboratory Animal Care of Shengjing Hospital. Briefly, GECs resuspended in 400 μL of solution containing 80% Matrigel at a density of 3 × 10^5^ cells per ml, and then it were subcutaneously injected. After 4 days, plugs were obtained. Weighed, photographed, and dispersed in 400 μL of PBS at 4 °C overnight incubation to collect the hemoglobin. Drabkin’s solution (Sigma) was conducted to measure hemoglobin content, according to manufacturer’s instructions.

### Statistical analysis

Experimental data were presented as mean ± standard deviation (SD) from at least three independent experiments. All statistical analyses were evaluated by SPSS 18.0 statistical software (IBM, New York, NY). Student’s t-test (two tailed) was used for comparisons between two groups. One-way analysis of variance (ANOVA) was used for multi-group comparisons followed by Bonferroni post-hoc analysis. The value of *P* < 0.05 was considered statistically significant.

## Results

### Knockdown of SRSF10 suppressed the proliferation, migration and tube formation of GECs

**Fig. 1:**
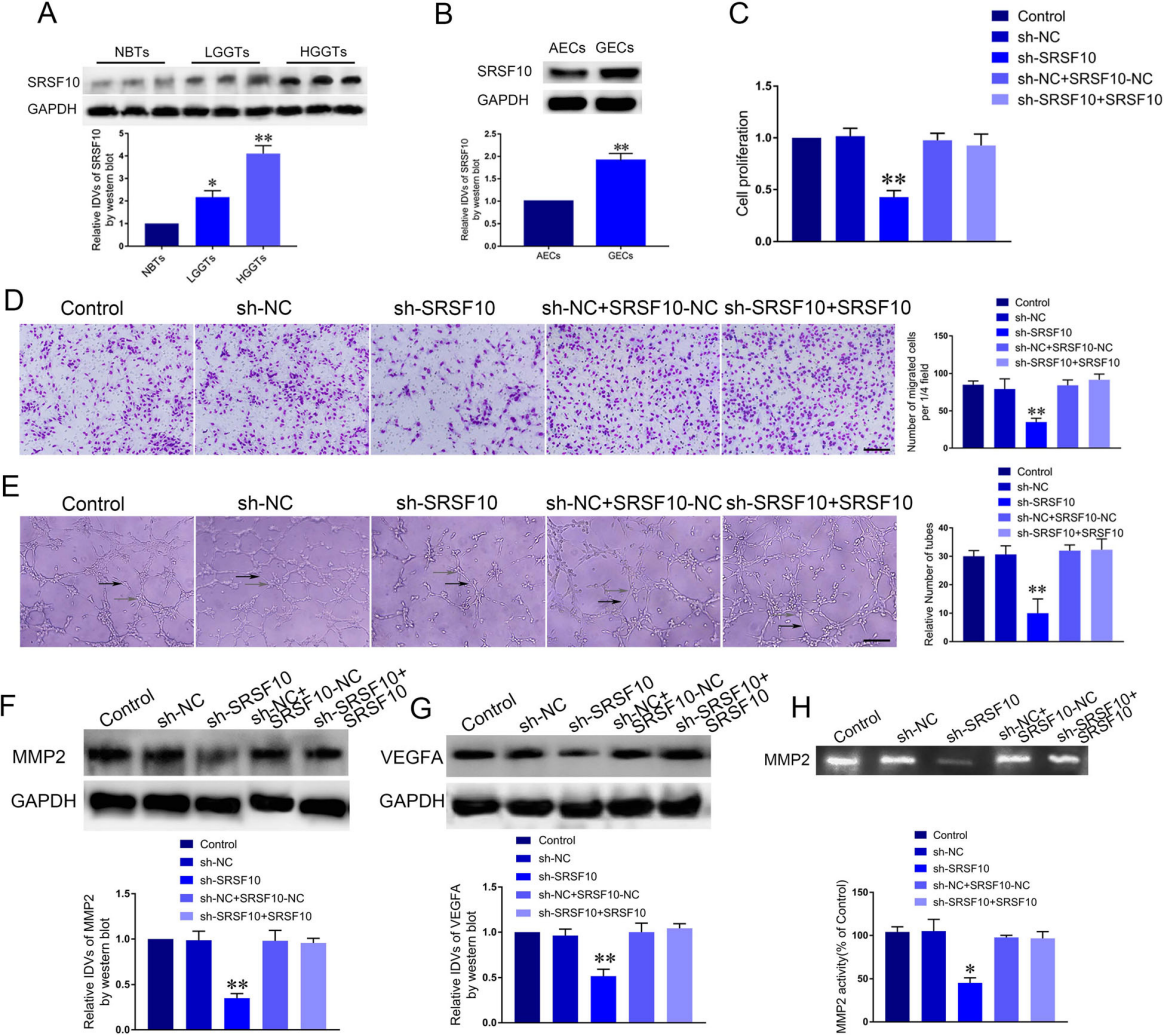
Silencing of SRSF10 affected the proliferation, migration and tube formation of GECs. **a** The relative protein expression of SRSF10 in NBTs and LGGTs, HGGTs were evaluated by Western-blot assay. GAPDH was used as an endogenous control. Data represent mean ± SD (*n* = 5, each group; **P < 0.01). **b** The relative protein expression of SRSF10 in AECs and GECs were evaluated by Western-blot assay. GAPDH was used as an endogenous control. Data represent mean ± SD (*n* = 3, each group; **P < 0.01). **c** The effect of SRSF10 on the viability of GECs was determined by CCK-8 assay. Data represent mean ± SD (*n* = 3, each group; **P < 0.01). **d** The effect of SRSF10 on the migration of GECs was assessed by Transwell assay. Data represent mean ± SD (*n* = 3, each group; **P < 0.01). Scale bar represents 30 μm. **e** The effect of SRSF10 on the tube formation of GECs was evaluated by Matrigel tube formation assay (Black arrow, tube structures and grey arrow, tube branches). Data represent mean ± SD (*n* = 3, each group; **P < 0.01). Scale bar represents 30 μm. **f** The effect of SRSF10 on the expression of MMP2. Data represent mean ± SD (*n* = 3, each group; **P < 0.01). **g** The effect of SRSF10 on the expression of VEGFA. Data represent mean ± SD (*n* = 3, each group; **P < 0.01). **h** The activity of MMP2 in the GECs after downregulation of SRSF10 was detected by gelatin zymography. Data represent mean ± SD (*n* = 3, each group; *P < 0.05)

RBPs are involved in the regulation of tumor cells’ biology process. To determine the functional role of RBPs in GECs, we first performed microarray to assess the expression patterns of RBPs in AECs and GECs. As shown in Supplementary Figure S1A, SRSF10 and RNPC1 are two of the most abundant RBPs in GECs. Subsequently, qRT-PCR was used to detect that expression of both SRSF10 and RNPC1 were elevated in GECs with the former being more evident (Supplementary Figure S1B). Further, we established GECs with downregulated SRSF10 to demonstrate the potential roles of SRSF10 in cell viability. Thus, SRSF10 was selected to perform the subsequent analyses. Further, the expression of SRSF10 protein was detected through western blot in brain tissues (normal brain tissues, NBTs), low-grad glioma tissues (LGGTs, Word Health Organization, grad I-II), high-grad glioma tissues HGGTs, Word Health Organization, grad III-IV), as well as in astrocyte-associated endothelial cells (AECs) and GECs. As shown in Fig. 1a, the expression of SRSF10 protein was upregulated in LGGTs group and HGGTs group compared with NBTs. Meanwhile, SRSF10 was significantly increased in GECs compared with AECs (Fig. 1b, *P* < 0.01). Then we demonstrated the impact of SRSF10 knockdown on the process of migration and tube formation in GECs. CCK-8 assay was used to detect cell proliferation. Interestingly, downregulation of SRSF10 decreased cell viability while SRSF10 overexpression on the basis of SRSF10 knockdown reversed the effect of SRSF10 knockdown on reducing cell viability (Fig. 1c). RNPC1 exerted no effects on cell viability (Supplementary Figure S1C). Transwell assay was conducted to assess migration. There was no significant difference in migration between Control group and sh-NC group. Migration of sh-SRSF10 group was significantly attenuated compared with sh-NC group. And SRSF10 overexpression on the basis of SRSF10 knockdown reversed the effect of SRSF10 knockdown on reducing migration (Fig. 1d, *P* < 0.01). Matrigel plug assay was conducted to detect tube formation with knockdown of SRSF10. Knockdown of SRSF10 inhibited the tube formation, while SRSF10 overexpression on the basis of SRSF10 knockdown reversed the effect of SRSF10 knockdown on inhibiting tube formation (Fig. 1e, *P* < 0.01). Subsequently, we assessed the effect of SRSF10 knockdown on MMP2 and VEGFA with western blot assay. The expression of MMP2 and VEGFA in sh-SRSF10 group were both significantly decreased compared with sh-NC group. SRSF10 overexpression on the basis of SRSF10 knockdown reversed the effect of SRSF10 knockdown on reducing MMP2 and VEGFA protein expression (Fig. 1f-g, *P* < 0.01). Meanwhile, to identify the gelatinolytic activity of MMP2, gelatin zymography was performed. The zymography studies demonstrated that the activity of MMP2 were markedly decreased in sh-SRSF10 group compared with sh-NC group, and SRSF10 overexpression on the basis of SRSF10 knockdown reversed the effect of SRSF10 knockdown on reducing the activity of MMP2. (Fig. 1h, *P* < 0.05). The above results proved SRSF10 knockdown lowered MMP2 expression and activity to inhibit GEC-induced angiogenesis in vitro.

### Knockdown of circ-ATXN1 suppressed the proliferation, migration and tube formation of GECs

**Fig. 2:**
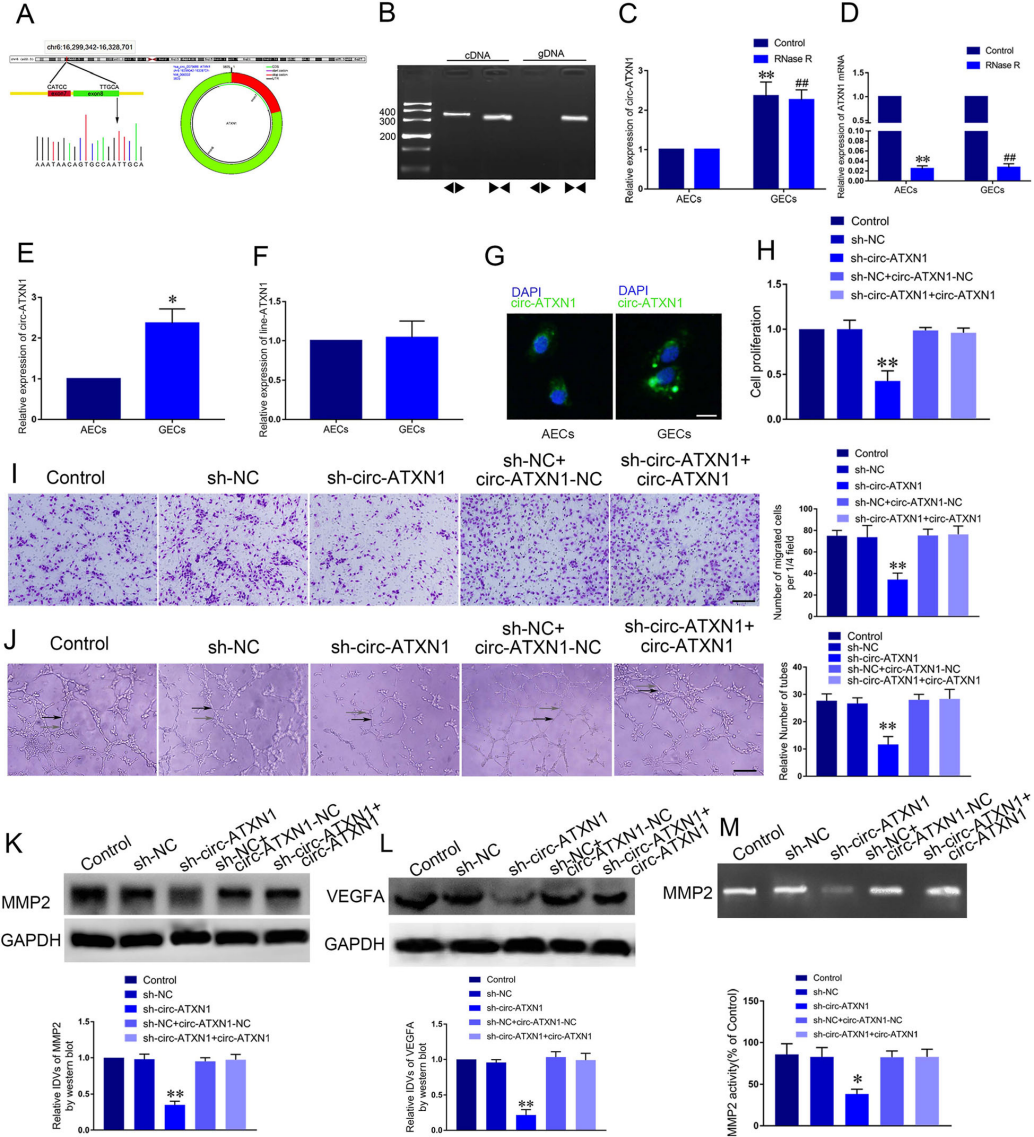
Silencing of circ-ATXN1 affected the proliferation, migration and tube formation of GECs. **a** A schematic representation of how circ-ATXN1 arosed from the ATXN1 gene as determined by scanning ATXN1 genomic DNA and circBase. Sanger sequencing validated the sequence on the junction sites of circRNA-ATXN1. The black arrow indicated the head-to-tail splicing sites of circRNA-ATXN1. **b** The existence of circ-ATXN1 in GECs. **c** The expression of circ-ATXN1 in GECs treated with RNase R. Data represent mean ± SD (*n* = 3, each group; **P < 0.01, ^##^*P* < 0.01). **d** The expression of line-ATXN1 in GECs treated with RNase R. Data represent mean ± SD (*n* = 3, each group; **P < 0.01, ^##^*P* < 0.01). **e** The relative expression of circ-ATXN1 in AECs and GECs was detected by qRT-PCR. GAPDH was used as an endogenous control. Data represent mean ± SD (*n* = 3, each group; *P < 0.05) **f** The relative expression of line-ATXN1 was detected in AECs and GECs by qRT-PCR. GAPDH was used as an endogenous control. Data represent mean ± SD (*n* = 3, each group). **g** FISH was performed to investigate expression and location of circ-ATXN1 in AECs and GECs (green, circ-ATXN1; blue, DAPI nuclear staining). **h** Effect of circ-ATXN1 on the cell viability of GECs was detected by CCK-8 assay. Data represent mean ± SD (*n* = 3, each group; **P < 0.01). **i** Effect of circ-ATXN1 on the migration of GECs was detected by Transwell assay. Data represent mean ± SD (*n* = 3, each group; **P < 0.01). **j** Effect of circ-ATXN1 on tube formation of GECs was measured by Matrigel tube formation assay (Black arrow, tube structures; Grey arrow, tube branches). Data represent mean ± SD (*n* = 3, each group, **P < 0.01). Scale bar represents 30 μm. **k**-**l** Effect of circ-ATXN1 knockdown on expression of MMP2 and VEGFA was detected by Western blot analysis. Data represent mean ± SD (*n* = 3, each group; **P < 0.01). **m** The activity of MMP2 in the GECs after downregulation of circ-ATXN1 was detected by gelatin zymography. Data represent mean ± SD (*n* = 3, each group; *P < 0.05)

Using circ-RNA microarray, we found circ-ATXN1 was significantly downregulated after knockdown of SRSF10 in GECs (Supplementary Figure S1D-E). Thus, we hypothesized that circ-ATXN1 was involved in SRSF10-mediated regulation on glioma angiogenesis. Figure 2a showed that the composition of circ-ATXN1 by presenting the genomic DNA sequence of ATXN1 in contrast to circ-ATXN1. The junction sites of circ-ATXN1 CATCCAGAGC and GCCAATTGCA, which were further validated by Sanger sequencing experiment. Subsequently, divergent primers detected the presence of circ-ATXN1 in cDNA while no circ-ATXN1 was detected in the genomic DNA (gDNA) (Fig. 2b). Then, qRT-PCR assay was used to verify that circ-ATXN1 was significantly increased in GECs compared with AECs, while there was no significant difference in linear-ATXN1 expression between GECs and AECs (Fig. 2e-f, *P* < 0.01). RNase R (an exoribonuclease that degrades linear RNAs instead of circRNAs) was applied to distinguish circ-ATXN1 from linear-ATXN1. Circ-ATXN1 exhibited resistance to RNase R, whereas linear-ATXN1 was degraded after treatment with RNase R (Fig. 2c-d). Thus, circ-ATXN1 instead of linear-ATXN1 may regulate the function of GECs. To investigate the functional role of circ-ATXN1, we further detected the location and expression of circ-ATXN1 by FISH in GECs and AECs and verified that circ-ATXN1 was located in the cytoplasm and its expression was increased in GECs (Fig. 2g). Subsequently, we established glioma cell lines with circ-ATXN1 knockdown or overexpression, then cultured ECs in glioma conditioned medium generated with the above-mentioned cell line. Cell viability, migration and tube formation were analyzed. As shown in Fig. 2h-j, the cell viability, migration and tube formation in sh-circ-ATXN1 group were significantly repressed compared with sh-NC group. Also, circ-ATXN1 overexpression on the basis of circ-ATXN1 knockdown reversed the effect of circ-ATXN1 knockdown on inhibition of cell viability, migration, and tubes formation. Besides, the expression of MMP2 and VEGFA as well as MMP2 activity were markedly attenuated after circ-ATXN1 knockdown. Overexpression of circ-ATXN1 reversed the reduced expression of MMP2 and VEGFA, and the activity of MMP2 (Fig. 2k-m, *P* < 0.01).

### SRSF10 bound to circ-ATXN1 in regulating cell viability, migration and tube formation of GECs

**Fig. 3:**
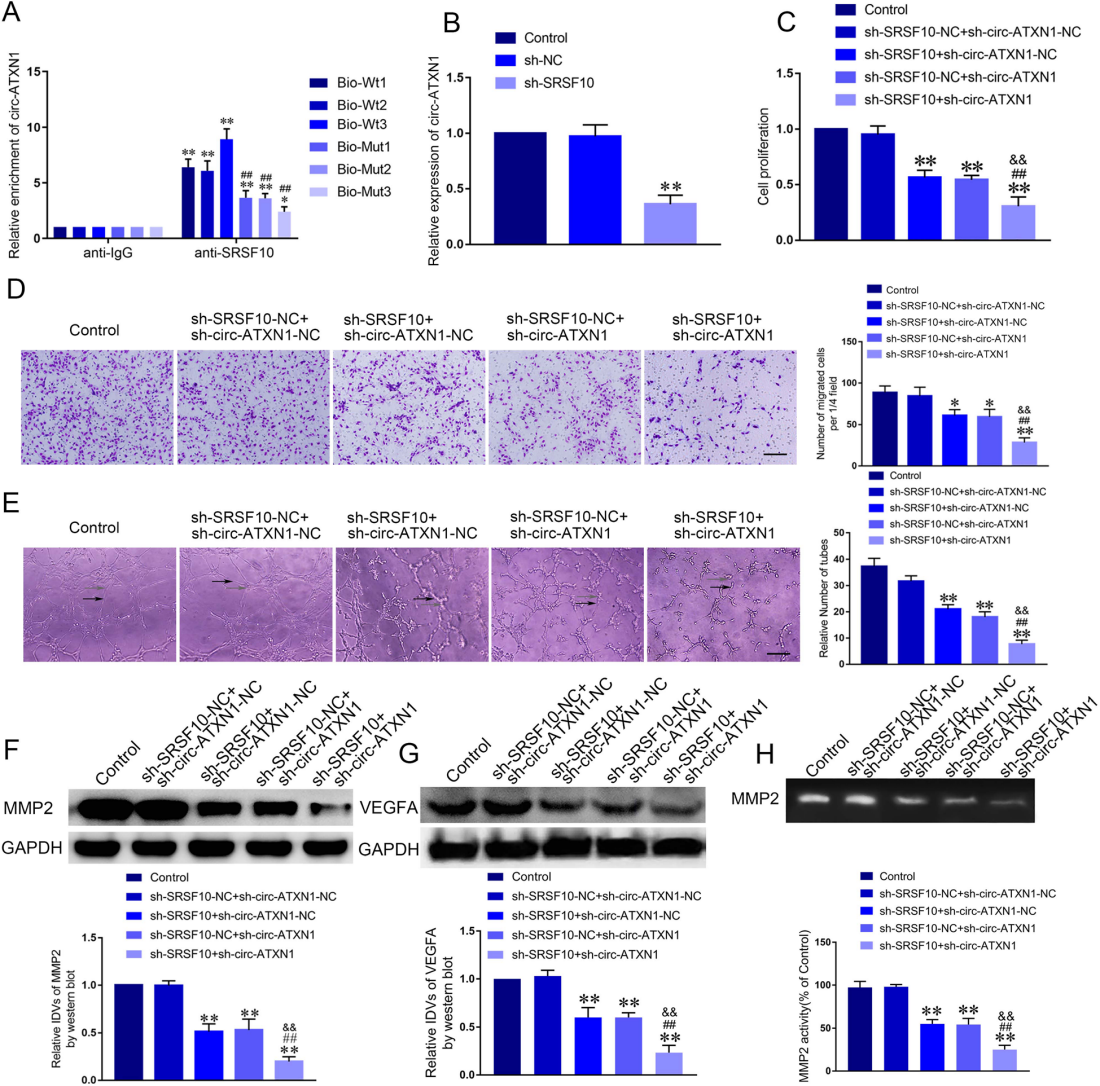
Circ-ATXN1 was involved in SRSF10-mediated angiogenesis of GECs. **a** Relative enrichment of circ-ATXN1 in anti-IgG and anti-SRSF10 were detected by RNA immunoprecipitation assay. Data represent mean ± SD (*n* = 3, each group; **P < 0.01, ^##^*P* < 0.01). **b** Effect of SRSF10 knockdown on expression of circ-ATXN1 was detected by qRT-PCR. Data represent mean ± SD (*n* = 3, each group; ** *P* < 0.01). **c** Effects of circ-ATXN1 and SRSF10 on the cell viability of GECs were detected by CCK-8 assay. Data represent mean ± SD (*n* = 3, each group; **P < 0.01, ^##^*P* < 0.01, ^&&^*P* < 0.01). **d** Effects of circ-ATXN1 and SRSF10 on the migration of GECs were detected by Transwell assay. Data represent mean ± SD (*n* = 3, each group; **P < 0.01, ^##^*P* < 0.01, ^&&^*P* < 0.01). **e** Effects of circ-ATXN1 and SRSF10 on tube formation of GECs were measured by Matrigel tube formation assay. (Black arrow, tube structures; Grey arrow, tube branches). Data represent mean ± SD (*n* = 3, each group; **P < 0.01, ^##^*P* < 0.01, ^&&^*P* < 0.01). Scale bar represents 30 μm. **f**-**g** Effects of circ-ATXN1 and SRSF10 on expressions of MMP2 and VEGFA were detected by western blot. Data represent mean ± SD (*n* = 3, each group; **P < 0.01, ^##^*P* < 0.01, ^&&^*P* < 0.01). **h** Effects of circ-ATXN1 and SRSF10 on activity of MMP2 were detected by gelatin zymography. Data represent mean ± SD (*n* = 3, each group; *P < 0.05)

In order to clarify the molecular mechanism of SRSF10 in regulating the angiogenesis of GECs, we conducted RIP analysis to determine the interaction between SRSF10 and circ-ATXN1. Enrichment of Bio-pre-circ-ATXN1-Wt3 in SRSF10 co-immunoprecipitation group was significantly elevated compared with IgG co-immunoprecipitation group. Enrichment of Bio-pre-circ-ATXN1-Wt3 in SRSF10 co-immunoprecipitation group was significantly decreased compared with Bio-pre-circ-ATXN1-Mut3 (Fig. 3a, *P* < 0.01). We subsequently assessed circ-ATXN1 expression after knockdown of SRSF10 using qRT-PCR. As shown in Fig. 3b, the expression of circ-ATXN1 in sh-SRSF10 group was significantly decreased. Cell lines with co-transfection of SRSF10 and cir-ATXN1 were established. Meanwhile, we detected that the cell viability of GECs in sh-SRSF10 + sh-circ-ATXN1 group was significantly attenuated compared with sh-SRSF10-NC + sh-circ-ATXN1-NC, sh-SRSF10 + sh-circ-ATXN1-NC and sh-SRSF10-NC + sh-circ-ATXN1-NC groups, respectively (Fig. 3c, *P* < 0.01). Migration and tube formation analysis yielded similar results (Fig. 3d-e, *P* < 0.01). The above results demonstrated simultaneous knockdown of circ-ATXN1 and SRSF10 intensified inhibition of cell viability, migration and tube formation induced by SRSF10 knockdown. Likewise, the expression of MMP2 and VEGFA, as well as the activity of MMP2 were significantly decreased in sh-SRSF10 + sh-circ-ATXN1 group (Fig. 3f-h, *P* < 0.01). The above results suggested simultaneous knockdown of SRSF10 and circ-ATXN1 inhibited the expression of MMP2 and VEGFA more significantly. We verified that circ-ATXN1, involved in the regulation of GECs angiogenesis, was regulated by SRSF10.

### Up-regulation of miR-526b-3p suppressed angiogenesis of GECs

**Fig. 4:**
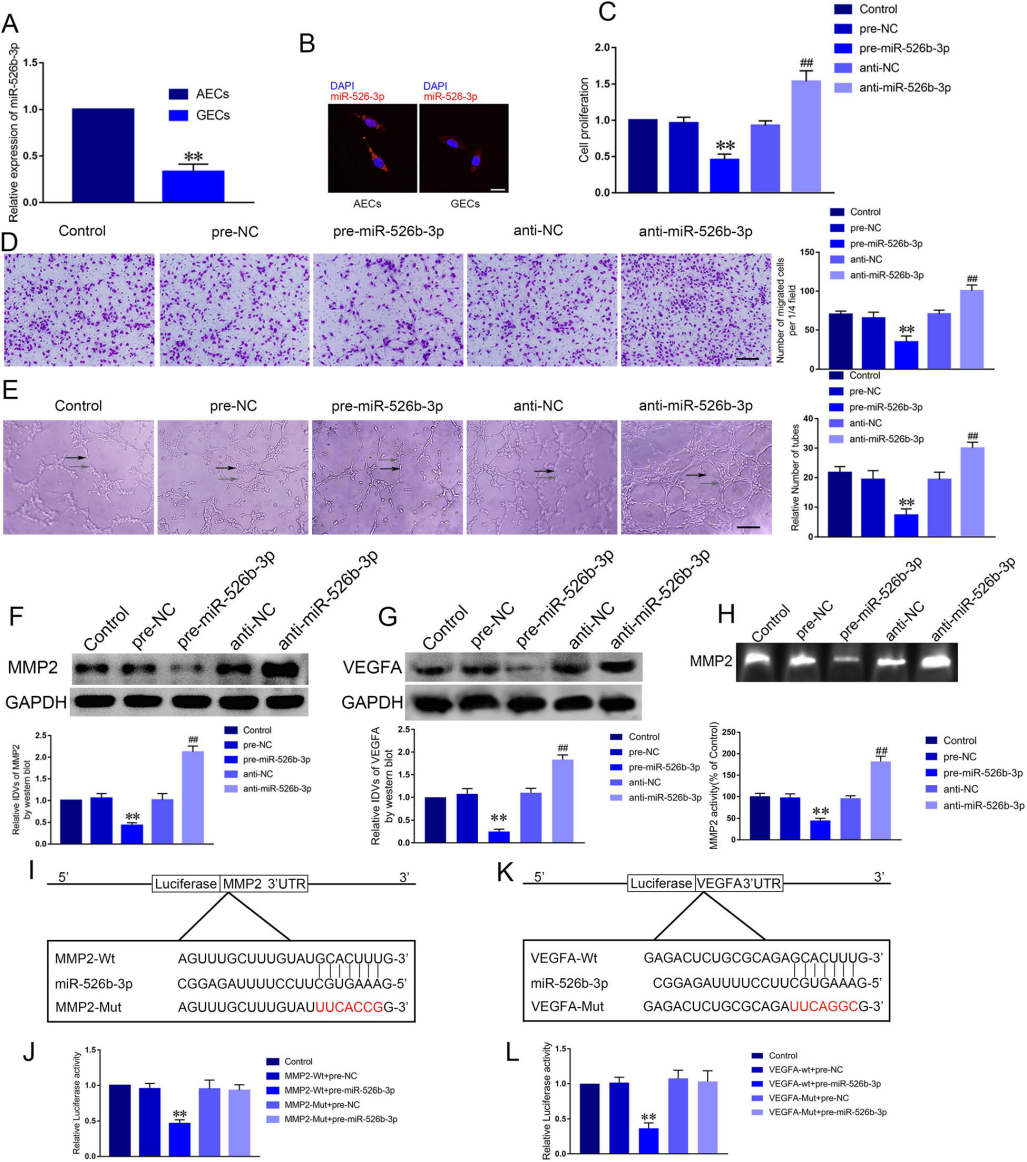
MiR-526b-3p affected the proliferation, migration and tube formation of GECs via binding to MMP2. **a** Relative expression levels of miR-526b-3p in AECs and GECs were determined by qRT-PCR. Data represent mean ± SD (*n* = 3, each group; **P < 0.01). **b** FISH was performed to investigate expression and location of miR-526b-3p in AECs and GECs (red, miR-526b-3p; blue, DAPI nuclear staining). **c** The effect of miR-526b-3p on the viability of GECs was determined by CCK-8 assay. Data represent mean ± SD (*n* = 3, each group; **P < 0.01, ^##^*P* < 0.01). **d** The effect of miR-526b-3p on the migration of GECs was assessed by Transwell assay. Data represent mean ± SD (*n* = 3, each group; **P < 0.01, ^##^*P* < 0.01). **e** The effect of miR-526b-3p on the tube formation of GECs was evaluated by Matrigel tube formation assay (Black arrow, tube structures; Grey arrow, tube branches). Data represent mean ± SD (*n* = 3, each group; **P < 0.01, ^##^*P* < 0.01). Scale bar represents 30 μm. **f**-**g** The effect of miR-526b-3p on the expression of MMP2 and VEGFA. Data represent mean ± SD (*n* = 3, each group; **P < 0.01, ^##^*P* < 0.01). **h** Effects of miR-526b-3p on the activity of MMP2. Data represent mean ± SD (*n* = 3, each group; **P < 0.01, ^##^*P* < 0.01). **i** The putative miR-526b-3p binding site in MMP2 (MMP2-Wt) and the designated mutant sequence (MMP2-Mut) were illustrated. **j** Luciferase reporter assay of HEK293T cells co-transfected with MMP2-Wt or MMP2-Mut and miR-526b-3p or miR-526b-3p-NC. Data represent mean ± SD (*n* = 3, each group; **P < 0.01). **k** The putative miR-526b-3p binding site in VEGFA (VEGFA -Wt) and the designated mutant sequence (VEGFA-Mut) were illustrated. **l** Luciferase reporter assay of HEK293T cells co-transfected with VEGFA -Wt or VEGFA -Mut and miR-526b-3p or miR-526b-3p-NC. Data represent mean ± SD (*n* = 3, each group; **P < 0.01)

Using microarray, we found miR-92a and miR-526b-3p were two of upregulated genes in GECs treated with sh-circ-ATXN1 (Supplementary Figure S1F). Meanwhile, qRT-PCR was used to assess that miR-526b-3p expression was higher than miR-92a in GECs treated with sh-circ-ATXN1 (Supplementary Figure 1G). Subsequently, we detected that miR-526b-3p was significantly decreased in GECs compared with AECs (Fig. 4a, *P* < 0.01). In addition, FISH was used to demonstrate that miR-526b-3p was located in cytoplasm and downregulated in GECs (Fig. 4b). In order to further clarify the effect of miR-526b-3p on regulating biological behaviors of GECs, we established glioma cell lines with overexpression and knockdown of miR-526b-3p. ECs were cultured in glioma conditioned medium generated with the above-mentioned cell lines. Cell viability, migration and tube formation were analyzed. The proliferation of pre-miR-526b-3p group was significantly inhibited compared with pre-NC group and the proliferation of anti-miR-526b-3p group was significantly elevated compared with anti-NC group (Fig. 4c, *P* < 0.01). Likewise, the migration and tube formation of pre-miR-526b-3p group were significantly inhibited compared with pre-NC group (*P* < 0.01). Anti-miR-526b-3p group yielded the opposite results compared with anti-NC group (Fig. 4d-e, *P* < 0.01). The above results indicated that miR-526b-3p suppressed angiogenesis of GECs.

### MiR-526b-3p regulated angiogenesis of GECs via specifically binding to MMP2 and VEGFA respectively

It has been widely reported that miRNAs specifically bound to 3’UTR of mRNAs to inhibit their translation or facilitate their degradation. Western blot assay was conducted to detect MMP2 and VEGFA expression with alteration of miR-526b-3p. In the present study, we measured that MMP2 and VEGFA expression of pre-miR-526b-3p group was significantly inhibited compared with pre-NC group and the expression of anti-miR-526b-3p group was significantly elevated compared with anti-NC group (Fig. 4f-g, *P* < 0.01). Likewise, the activity of MMP2 in pre-miR-526b-5p group was significantly reduced compared with pre-NC, while MMP2 activity was increased in anti-miR-526b-3p group (Fig. 4h). We predicted 3’UTR of MMP2 and VEGFA harbored binding sites with miR-526b-3p respectively using Targetscan (http://www.targetscan.org/vert_72/) (Fig. 4i and k). Next, dual-luciferase reporter analysis demonstrated firefly/renilla luciferase activity of MMP2–3′-UTR-Wt and miR-526b-3p (+) co-transfection group was significantly decreased compared with MMP2–3′-UTR-Wt + pre-NC group. Similar results were observed in VEGFA (Fig. 4j and l). In summary, miR-526-3p affected angiogenesis of GECs via regulating the expression of MMP2 and VEGFA.

### Circ-ATXN1 promoted angiogenesis of GECs via binding to miR-526b-3p

**Fig. 5:**
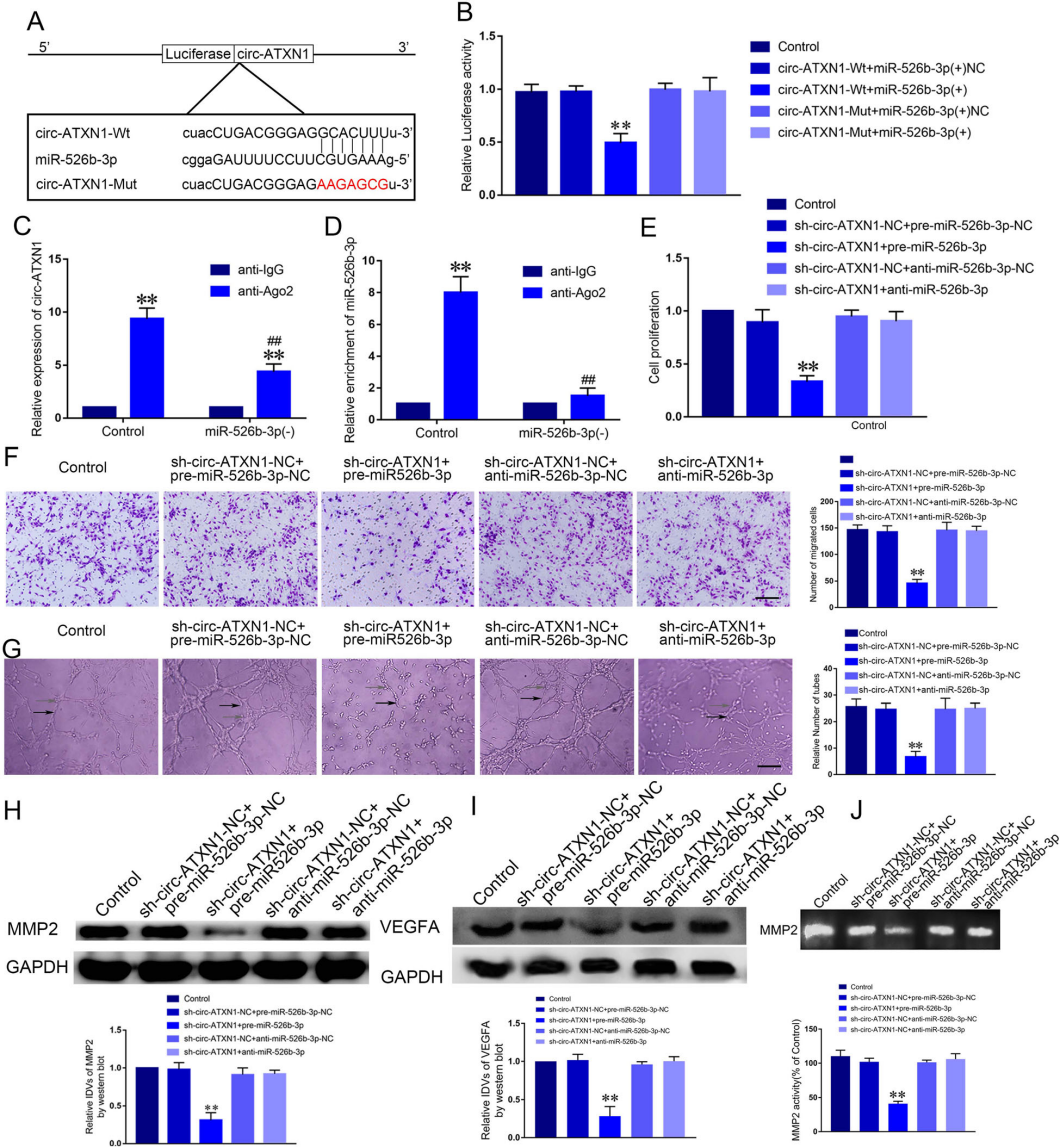
Circ-ATXN1 facilitated angiogenesis of GECs via binding to miR-526b-3p. **a** The putative miR-526b-3p binding site in circ-ATXN1 (circ-ATXN1-Wt) and the designated mutant sequence (circ-ATXN1-Mut) were illustrated. **b** Luciferase reporter assay of HEK293T cells co-transfected with circ-ATXN1-Wt or circ-ATXN1-Mut and miR-526b-3p or miR-526b-3p-NC. Data represent mean ± SD (*n* = 3, each group; **P < 0.01). **c**-**d** The interaction between circ-ATXN1 and miR-526b-3p was determined by RIP assay. Circ-ATXN1 expression and miR-526b-3p enrichment were measured using real-time qPCR. Data represent mean ± SD (*n* = 3, each group; **P < 0.01, ^##^*P* < 0.01). **e** The co-effects of circ-ATXN1 and miR-526b-3p on the viability of GECs were evaluated by CCK-8 assay. **f** The co-effects of circ-ATXN1 and miR-526b-3p on the migration of GECs were evaluated by Transwell assay. **g** The co-effects of circ-ATXN1 and miR-526b-3p on the tube formation of GECs were evaluated by Matrigel tube formation assay (Black arrow, tube structures; Grey arrow, tube branches). Data are presented as the mean ± SD (*n* = 3, each group; **P < 0.01 vs sh-circ-ATXN1-NC + pre-miR-526b-3p group. Scale bar represents 30 μm). **h**-**i** The co-effects of circ-ATXN1 and miR-526b-3p on the expression of MMP2 and VEGFA. Data represent mean ± SD (*n* = 3, each group; **P < 0.01). **j** The co-effects of circ-ATXN1 and miR-526b-3p on the activity of MMP2. Data represent mean ± SD (*n* = 3, each group; **P < 0.01)

The existence of targeted binding between circ-ATXN1 and miR-526-3p were predicted using Starbase (Fig. 5a), thus we presumed that circ-ATXN1 regulated angiogenesis of GECs via regulating miR-526-3p. Dual-luciferase reporter analysis was conducted to verify the binding between circ-ATXN1 and miR-526-3p. Luciferase activity of circ-ATXN1-Wt and miR-526b-3p(+) co-transfection group was significantly decreased compared with circ-ATXN1-Wt + miR-526b-3p(+)NC group, suggesting circ-ATXN1 specifically bound to the predicted site of miR-526-3p (Fig. 5b, *P* < 0.01). RIP analysis was conducted to determine whether circ-ATXN1 and miR-526-3p were in the expected Ago2 immunoprecipitation complex. Expression of circ-ATXN1 and miR-526-3p in Ago2 co-immunoprecipitation group were significantly increased compared with IgG co-immunoprecipitation group (*P* < 0.01). Knockdown of miR-526-3p significantly lowered the enrichment of circ-ATXN1 and miR-526-3p in Ago2 co-immunoprecipitation groups (Fig. 5c-d, *P* < 0.01). These results suggested that circ-ATXN1 and miR-526-3p were in the expected Ago2 immunoprecipitation complex. We established glioma cell lines stably expressing circ-ATXN1 knockdown with up-regulation and down-regulation of miR-526-3p. ECs were cultured in glioma conditioned medium generated with the above-mentioned cell lines. Knockdown of miR-526-3p reversed the decreased proliferation, migration and tube formation of GECs induced by knockdown of circ-ATXN1 (Fig. 5e-g, *P* < 0.01). The down-regulated expression of MMP2 and VEGFA induced by knockdown of circ-ATXN1 was reversed after knockdown of miR-526b-3p (Fig. 5h-i, *P* < 0.01). Meanwhile, the activity of MMP2, which was decreased after knockdown of circ-ATXN1, was reversed by silencing miR-526b-3p (Fig. 5j). The above results demonstrated that miR-526-3p was involved in the regulation of GECs angiogenesis by circ-ATXN1. MiR-526-3p was also critical in the regulation of MMP2 and VEGFA by circ-ATXN1. In this study, we further investigated the effects of co-transfection of knockdown of SRSF10 and knockdown of miR-526b-3p. The results showed that, knockdown of miR-526b-3p reversed the effects of SRSF10 knockdown on the proliferation, migration and tube formation of GECs, as well as the expression of MMP2, VEGFA, and the activity of MMP2 (Supplementary Figure S1H-L).

### SRSF10 knockdown combined with circ-ATXN1 knockdown and miR-526b-3p overexpression suppressed glioma angiogenesis in vivo

**Fig. 6:**
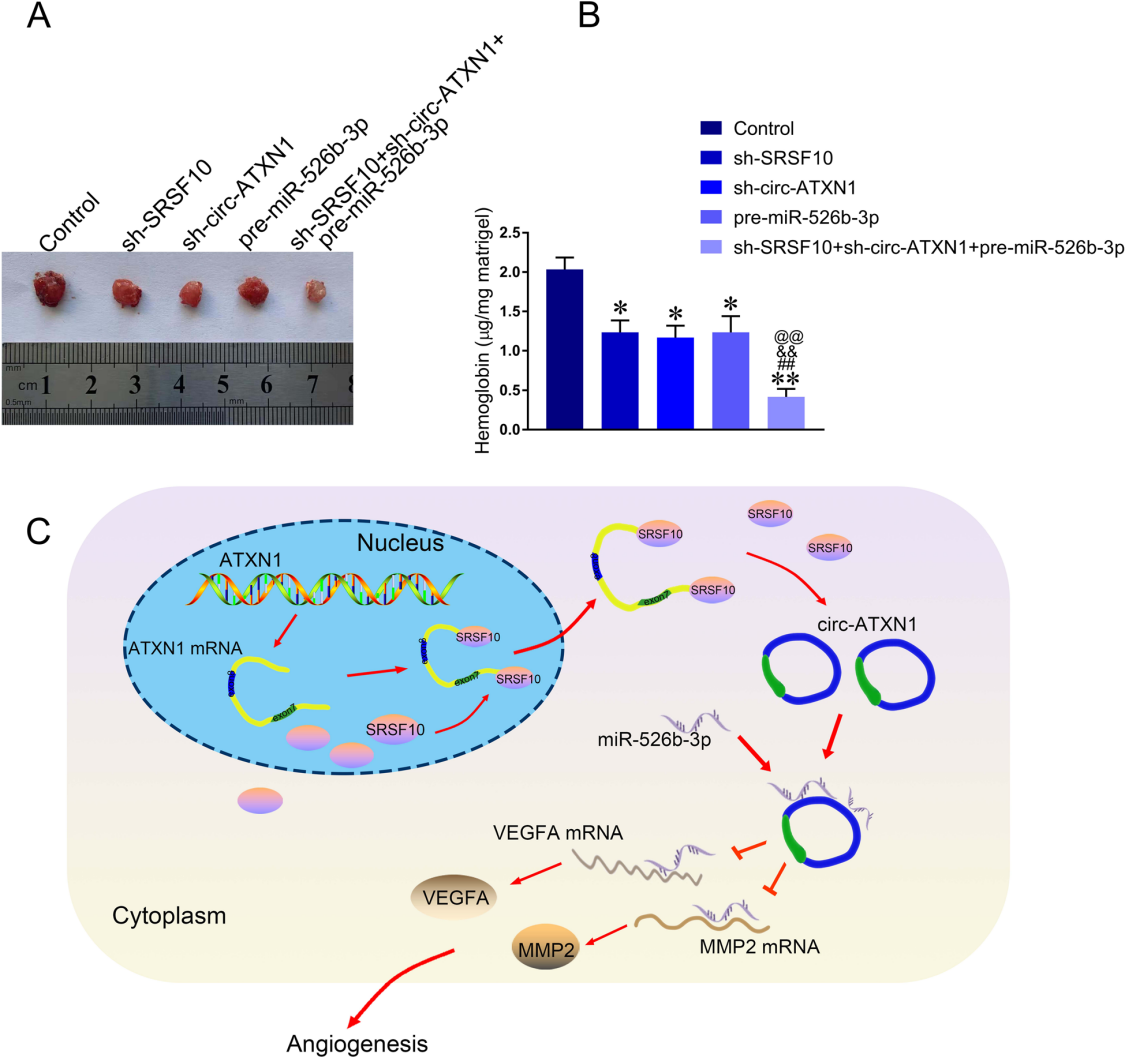
SRSF10 knockdown combined with circ-ATXN1 knockdown, and miR-256-3p overexpression suppressed the angiogenesis in vivo. **a** Matrigel plug assay was used to measure angiogenesis. **b** The hemoglobin content was measured. Data are presented as mean ± SD (*n* = 3, each group), *P < 0.05, **P < 0.01vs. Control group; ^##^*P* < 0.01 vs. sh-SRSF10 group; ^&&^*P* < 0.01 vs. sh-circ-ATXN1 group; ^@@^*P* < 0.01 vs. pre-miR-526-3p group. **c** Schematic illustration of the mechanism of SRAF10/circ-ATXN1/miR-526b-3p regulating glioma angiogenesis

In our study, Matrigel plug assay was conducted to assess whether SRSF10 and circ-ATXN1 were able to regulate the angiogenesis of GECs in vivo. As shown in Fig. 6a-b, the hemoglobin content in sh-SRSF10 group, sh-circ-ATXN1 group, and pre-miR-526b-3p group, and sh-SRSF10 + sh-circ-ATXN1 + pre-miR-526b-3p group were decreased compared with Control group (*P* < 0.05). Meanwhile, the hemoglobin content in sh-SRSF10 + sh-circ-ATXN1 + pre-miR-526b-3p group was significantly decreased compared with sh-SRSF10 group, sh-circ-ATXN1 group, and pre-miR-526b-3p group respectively. Thus, the above results indicated that the combination of SRSF10 knockdown, circ-ATXN1 knockdown and miR-526b-3p overexpression presented the strongest inhibitory effect on glioma angiogenesis in vivo.

## Discussion

In the present study, we verified that SRSF10 and circ-ATXN1 were both increased in GECs. Knockdown of SRSF10 and circ-ATXN1 suppressed proliferation, migration and tube formation of GECs, respectively. SRSF10 bound to the 5′-end and 3′-end of circ-ATXN1 pre-mRNA and promoted circ-ATXN1 biogenesis. In addition, we demonstrated that miR-526b-3p was decreased and exerted anti-tumor functions in GECs. Furthermore, we indicated that circ-ATXN1 bound to miR-526b-3p and inhibited GECs angiogenesis by inhibiting miR-526b-3p expression. In summary, we discovered the molecular regulatory network in which SRSF10 facilitated circ-ATXN1 biogenesis and subsequently increased its binding with miR-526b-3p to inhibit the negative regulatory effect of miR-526b-3p on MMP2 and VEGFA in regulating glioma angiogenesis.

RBPs mediate the maturation, transport, localization and translation of RNAs, acting as a key regulator of transcription and post-transcription. SRSF10 acts as an oncogene in multiple cancers. As a splicing factor, SRSF10 mediates IL1RAP’s alternative splicing and promotes oncogenesis in cervical cancer [31]. Moreover, SRSF10 promotes the splicing of transcription factor BCLAF1 to facilitate the tumorigenesis of colon cancer [14]. As expected, our results indicated that the expression of SRSF10 was elevated in GECs. However, the potential regulatory mechanism of SRSF10 is yet to be revealed. Multiple RBPs have been reported to regulate biological behaviors of vascular endothelial cells. RBP-LRR, RBP-EKL, and RBP-SSF both have been indicated to activate vascular endothelial growth factor (VEGF) to promote proliferation, migration and tube formation of human umbilical vein endothelial cells (HUVECs), suggesting their potential role as wound healing agents [32]. Transcription factor RBP-J mediated Notch receptors represses proliferation of endothelial cells to maintain vascular homeostasis in adult mice. Knockdown of RBP-J induces the spontaneous angiogenesis in multiple tissues [33]. Our study further verified that knockdown of SRSF10 suppressed the proliferation, migration and tube formation of GECs.

CircRNAs are 3′-5′ covalently closed RNA rings, with stable expression and specificity in tissues and diseases, thereby becoming potential biomarkers for the diagnosis and treatment of various diseases. Abnormalities in circRNA expression play important roles in tumorigenesis and development. Recent research study demonstrated that circ-DICER1 regulated proliferation, migration and tube formation of GECs through targeting MOV10 [34]. Accordingly, we screened out the circRNAs which were regulated by SRSF10 in GECs, and finally focused on circ-ATXN1. Notably, circ-ATXN1 expression was intact after being treated with RNase R to degrade ATXN1. Hence, we further determined that circ-ATXN1, rather than linear ATXN1, exerted effects on glioma angiogenesis. Moreover, we examined the expression and location of circ-ATXN1, and the detailed functions in GECs that exerted by circ-ATXN1. Our results indicated that knockdown of circ-ATXN1 suppressed the proliferation, migration and tube formation of GECs. In addition, circ-ATXN1 overexpression on the basis of circ-ATXN1 knockdown reversed the reduced proliferation, migration and tube formation. Various circRNAs have been reported to regulate biological behaviors of tumor and vessel endothelial cells. For example, circ_0001368 suppresses the progression of gastric cancer via miR-6506-5p/FOXO3 axis [35]. Circ_104916 is down-regulated in colorectal cancer. Overexpression of circ_104916 inhibits migration and invasion of colorectal cancer cells via repressing epithelial to mesenchymal transition (EMT) [36]. Wang J et al. discovered six circRNAs related to apoptosis, which induced abdominal aortic aneurysm via promoting endothelial cells growth from circRNA-miRNA networks [37]. Circ_0005015 facilitates retinal endothelial angiogenesis by regulating proliferation, migration and tube formation of endothelial cells [38]. Circ_0003575 is up-regulated in oxLDL induced HUVECs and regulates the proliferation and angiogenesis of HUVECs [39].

It has been widely reported that multiple RBPs can regulate the back-splicing and biogenesis of circRNAs [8, 40]. 90% of human circRNAs are predicted to harbor reverse complementary repeats in flanking introns, mainly in the form of Alu elements [41]. Nevertheless, RBPs can bind to the flanking introns of pre-mRNA (containing Alu elements) to induce the covalent binding between its 5′ end and 3′ end, facilitating the biogenesis of circRNAs. For example, ZKSCAN1, HIPK3 and EPHB4 promote circularization of pre-mRNAs to form circRNAs by recognizing the Alu elements on both flanking of pre-mRNAs [42]. Our study revealed that SRSF10 promoted the biogenesis of circ-ATXN1 by binding to Alu elements on both flanking of its pre-mRNA. Besides, knockdown of SRSF10 attenuated proliferation, migration and tube formation of GECs by suppressing the biogenesis of circ-ATXN1.

CircRNAs are widely involved in regulating tumorigenesis and biological behaviors of endothelial cells by binding to miRNAs. In the present study, we first verified the expression and location of miR-526b-3p in GECs. Overexpression of miR-526b-3p disrupted angiogenesis of GECs, whereas knockdown of miR-526b-3p produced the opposite effect. The results above demonstrated the role of miR-526b-3p as a tumor suppressor in GECs. Interestingly, circRNAs have been discovered to function as the molecular sponge of miRNAs via acting as their competing endogenous RNAs (ceRNAs). For instance, circ_0005015 elevates the expression of MMP-2, XIAP, and STAT3 thereby promoting the development of diabetes retinopathy via acting as a molecular sponge of miR-519d-3p [38]. Knockdown of circRNA_0059655, a sponge of miR-338-3p, inhibits the proliferation, migration and invasion of salivary adenoid cystic carcinoma cells [43]. Circ_001783 is overexpressed in breast cancer cells as well as tissues and promotes breast cancer progression via sponging miR-200c-3p [44]. Circ_0010729 is up-regulated in hypoxia-induced HUVEC and facilitates the proliferation of HUVEC, whereas suppressing its apoptosis by targeting the miR-186/HIF-1α axis [45]. In our study, we verified that circ-ATXN1 expression was negatively correlated with miR-526b-3p. The two factors exhibited opposite functional roles in regulating GECs angiogenesis. Meanwhile, we predicted the existence of binding sites between circ-ATXN1 and miR-526b-3p with the help of bioinformatics software. Meanwhile, dual-luciferase reporter assay was used to demonstrate the targeted binding between miR-526b-3p and circ-ATXN1. Moreover, RIP assay revealed circ-ATXN1 and miR-526b-3p interacted in an RNA-induced silencing complex (RISC). Knockdown of circ-ATXN1 combined with overexpression of miR-526b-3p significantly attenuated proliferation, migration and tube formation of GECs. Simultaneous knockdown of circ-ATXN1 and miR-526b-3p rescued the adverse effect on GECs angiogenesis induced by knockdown of circ-ATXN1. More importantly, knockdown of circ-ATXN1 combined with miR-526b-3p significantly reduced the hemoglobin content on glioma angiogenesis in vivo. These results suggested circ-ATXN1 regulated glioma angiogenesis via sponging miR-526b-3p.

Numerous researches have revealed that miRNAs could regulate the transcription of downstream genes via binding to 3’UTR of transcriptional factors. For example, miR-367-3p is down-regulated in glioma and binds to 3’UTR of CEBPA to suppresse its expression, subsequently inhibiting the proliferation, migration and invasion of glioma cells [46]. MMPs and VEGF have been reported to play various roles in cancer, inflammation, tissue remodeling and photoaging [47], regulating the expression level of mRNA as well as activation of their latent zymogen form. Additionally, alterations in MMPs expression and activity occur in tumor biology processes [48]. Recent study shows that miR-29b negatively regulates MMP2 expression and activity to suppress gastric cancer cell migration and tumor growth [49]. In addition, lncRNA PVT1 promotes angiogenesis via STAT3/VEGFA axis in gastric cancer [50]. In this study, we demonstrated that miR-526b-3p regulated the expression of MMP2 and VEGFA as well as the activity of MMP2. Moreover, MMP2 expression is disrupted in ischemia/reperfusion injury models, thereby leading to contractile dysfunction [51]. MMP2 promotes proliferation of hepatic stellate cells via ERK1/2 and PI3K signaling pathways during hepatic injury [52]. In our study, we verified that miR-526b-3p exhibited post-transcriptional regulatory effects on MMP2 and VEGFA expression by binding to its 3’UTR via dual-luciferase reporter assay, and in the meantime, miR-526b-3p suppressed glioma angiogenesis by specifically targenting and suppressing MMP2 and VEGFA. The negative regulation of target genes by miRNAs affects glioma angiogenesis. In HUVECs, miR-106b-5p binds to 3’UTR of Angpt2 to inhibit it transcription. Knockdown of miR-106b-5p facilitates angiogenesis by promoting Angpt2 expression [53].

## Conclusion

In conclusion, our study for the first time verified SRSF10 and circ-ATXN1 were increased in GECs, whereas miR-526b-3p was decreased in GECs. Knockdown of SRSF10 and circ-ATXN1 suppressed proliferation, migration and tube formation of GECs, respectively. MiR-526b-3p inhibited glioma angiogenesis via repressing MMP2 and VEGFA. Knockdown of SRSF10 promoted the negative regulatory effect of miR-526b-3p on MMP2 and VEGFA by inhibiting the biogenesis of circ-ATXN1, subsequently attenuating its function by targeting miR-526b-3p. As a result, knockdown of SRSF10 suppressed proliferation, migration and tube formation of GECs to inhibit glioma angiogenesis. The mechanism was schematically illustrated in Fig. 6c. This study elaborated the critical regulatory effects of SRSF10/circ-ATXN1/miR-526b-3p pathway on glioma angiogenesis. The above results established a new theory and experimental basis for anti-angiogenesis treatment for glioma.

## Supplementary information

**Additional file 1: Supplementary Figure S1.** The screening of SRSF10, circ-ATXN1, and miR-526b-3p, as well as the effects of co-transfection between SRSF10 and miR-526b-3p in GECs. (A)Microarray analysis was detected in AECs and GECs. Red indicates high relative expression and green indicates low relative expression. (B)Relative expression level of SRSF10, and RNPC1 determined by qRT-PCR. Data represent mean ± SD (*n* = 3, each group; *P < 0.05, **P < 0.01). (C). The effect of cell viability after knockdown RNPC1. Data represent mean±SD (n=3,each). (D) Microarray analysis was detected after downregulation of SRSF10 in GECs. Red indicates high relative expression and green indicates low relative expression. (E)Relative expression level of circ-SYNJ2, and circ-ATXN1 determined by qRT-PCR. Data represent mean ± SD (*n* = 3, each group; *P < 0.05, **P < 0.01). (F) Microarray analysis was detected after downregulation of circ-ATXN1 in GECs. Red indicates high relative expression and green indicates low relative expression. (G)Relative expression level of miR-92a, and miR-526b-3p determined by qRT-PCR. Data represent mean ± SD (*n* = 3, each group; *P < 0.05, **P < 0.01). (H) The effect of co-transfection between SRSF10 and miR-526b-3p on the viability of GECs was determined by CCK-8 assay. Data represent mean ± SD (*n* = 3, each group; **P < 0.01, ^##^*P* < 0.01). (I) The effect of co-transfection between SRSF10 and miR-526b-3p on the migration of GECs was assessed by Transwell assay. Data represent mean ± SD (n = 3, each group; **P < 0.01, ^##^*P* < 0.01). Scale bar represents 30 μm. (J) The effect of co-transfection between SRSF10 and miR-526b-3p on the tube formation of GECs was evaluated by Matrigel tube formation assay (Black arrow, tube structures and grey arrow, tube branches). Data represent mean ± SD (*n* = 3, each group; *P < 0.01, ^##^*P* < 0.01). Scale bar represents 30 μm. (K) The effect of co-transfection between SRSF10 and miR-526b-3p on the expression of MMP2 and VEGFA. Data represent mean ± SD (*n* = 3, each group; **P < 0.01, ^##^*P* < 0.01). (L) The activity of MMP2 in the GECs after downregulation of SRSF10 was detected by gelatin zymography. Data represent mean ± SD (*n* = 3, each group; *P < 0.05, ^##^*P* < 0.01).

**Additional file 2: Supplementary Figure S2.** Transfection efficiency of SRSF10, circ-ATXN1, and miR-526b-3p, as well as the effects of cell viability of RNPC1. A. Relative expression of SRSF10 in GECs by qRT-PCR. Data represent mean±SD (n=3,each). **P<0.01 vs. sh-NC group. B. Relative expression of circ-ATXN1 in GECs by qRT-PCR. Data represent mean±SD (n=3,each). **P<0.01 vs. sh-NC group. C. Relative expression of miR-526b-3p in GECs by qRT-PCR. Data represent mean±SD (n=3,each). **P<0.01 vs. pre-NC group, ^##^*P*<0.05 vs. anti-NC group. D. The effect of cell viability after knockdown RNPC1. Data represent mean±SD (n=3,each).

## Data Availability

The data that support the findings of this study are available on request from the corresponding author. The data are not publicly available due to privacy or ethical restrictions.

## Abbreviations

RBP: RNA binding protein
circRNA: Circular RNA
CCK-8: Cell counting kit-8
3′-UTR: 3′-untranslated region
GEC: Glioma associated endothelial cells
miRNA: MicroRNA
qRT-PCR: quantitative real-time PCR
RNA-IP: RNA-binding protein immunoprecipition
TSS: Transcription start site
HEK293T: Human embryonic kidney 293 T
