# Supplementary figures and images for: SRSF10 inhibits biogenesis of circ-ATXN1 to regulate glioma angiogenesis via miR-526b-3p/MMP2 pathway
Source: J Exp Clin Cancer Res. 2020 Jun 29;39:121. doi: 10.1186/s13046-020-01625-8 (PMC7325155; doi:10.1186/s13046-020-01625-8)

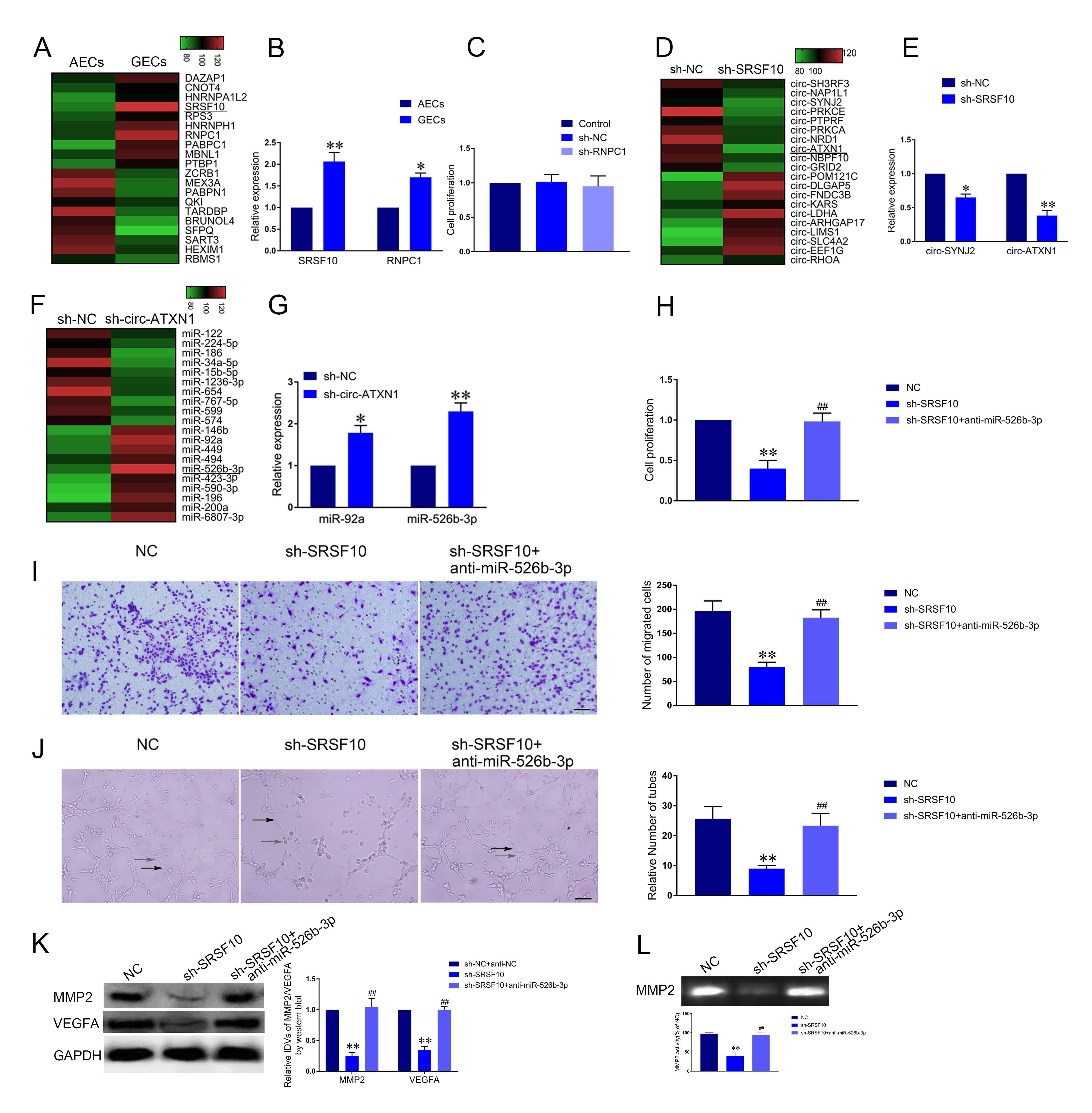

Supplement: Supplementary file 1 — Additional file 1: Supplementary Figure S1. The screening of SRSF10, circ-ATXN1, and miR-526b-3p, as well as the effects of co-transfection between SRSF10 and miR-526b-3p in GECs. (A)Microarray analysis was detected in AECs and GECs. Red indicates high relative expression and green indicates low relative expression. (B)Relative expression level of SRSF10, and RNPC1 determined by qRT-PCR. Data represent mean ± SD (n = 3, each group; *P < 0.05, **P < 0.01). (C). The effect of cell viability after knockdown RNPC1. Data represent mean±SD (n=3,each). (D) Microarray analysis was detected after downregulation of SRSF10 in GECs. Red indicates high relative expression and green indicates low relative expression. (E)Relative expression level of circ-SYNJ2, and circ-ATXN1 determined by qRT-PCR. Data represent mean ± SD (n = 3, each group; *P < 0.05, **P < 0.01). (F) Microarray analysis was detected after downregulation of circ-ATXN1 in GECs. Red indicates high relative expression and green indicates low relative expression. (G)Relative expression level of miR-92a, and miR-526b-3p determined by qRT-PCR. Data represent mean ± SD (n = 3, each group; *P < 0.05, **P < 0.01). (H) The effect of co-transfection between SRSF10 and miR-526b-3p on the viability of GECs was determined by CCK-8 assay. Data represent mean ± SD (n = 3, each group; **P < 0.01, ##P < 0.01). (I) The effect of co-transfection between SRSF10 and miR-526b-3p on the migration of GECs was assessed by Transwell assay. Data represent mean ± SD (n = 3, each group; **P < 0.01, ##P < 0.01). Scale bar represents 30 μm. (J) The effect of co-transfection between SRSF10 and miR-526b-3p on the tube formation of GECs was evaluated by Matrigel tube formation assay (Black arrow, tube structures and grey arrow, tube branches). Data represent mean ± SD (n = 3, each group; *P < 0.01, ##P < 0.01). Scale bar represents 30 μm. (K) The effect of co-transfection between SRSF10 and miR-526b-3p on the expression of MMP2 and VEGFA. Data re [file 13046_2020_1625_MOESM1_ESM.jpg]

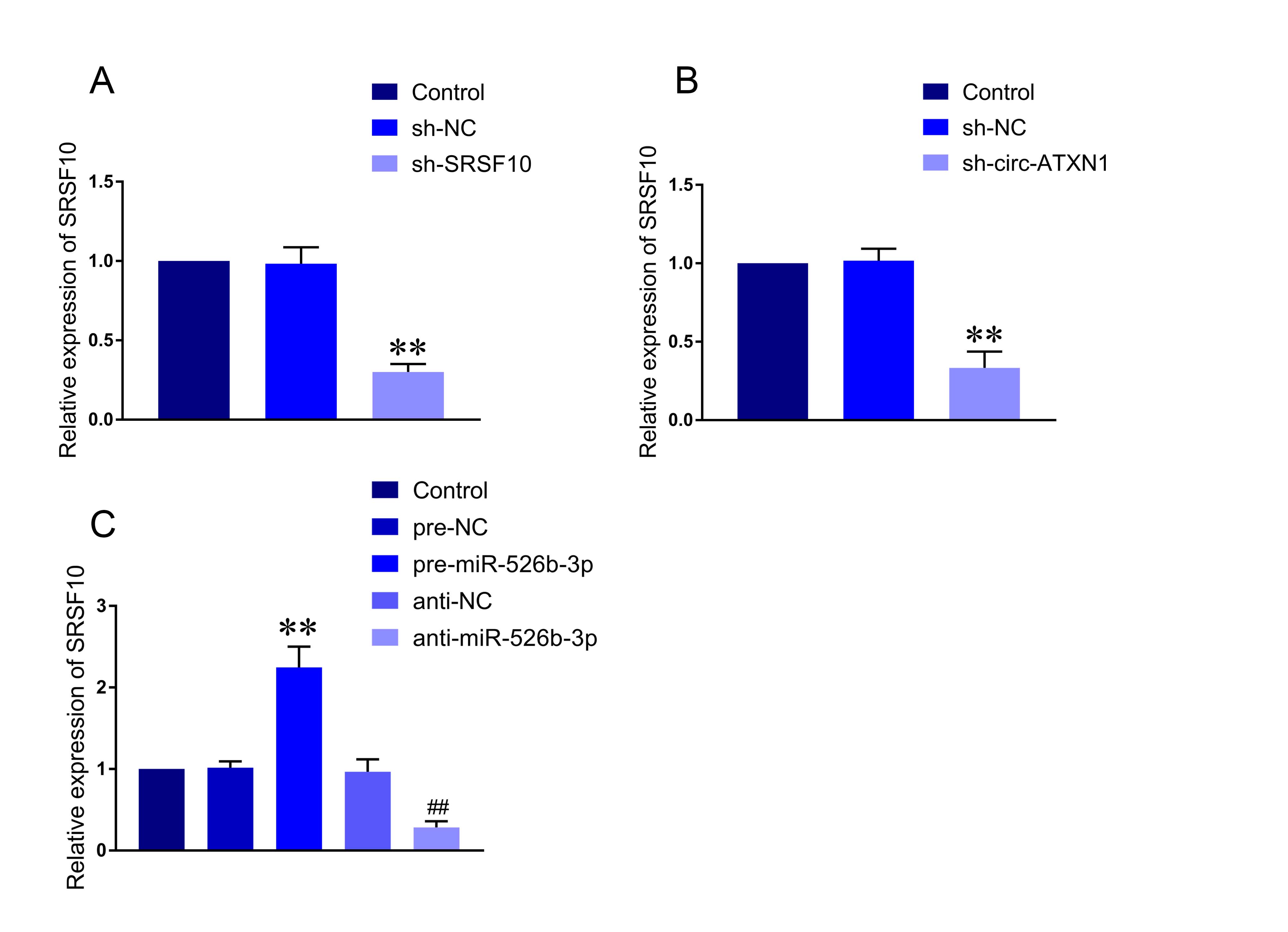

Supplement: Supplementary file 2 — Additional file 2: Supplementary Figure S2. Transfection efficiency of SRSF10, circ-ATXN1, and miR-526b-3p, as well as the effects of cell viability of RNPC1. A. Relative expression of SRSF10 in GECs by qRT-PCR. Data represent mean±SD (n=3,each). **P<0.01 vs. sh-NC group. B. Relative expression of circ-ATXN1 in GECs by qRT-PCR. Data represent mean±SD (n=3,each). **P<0.01 vs. sh-NC group. C. Relative expression of miR-526b-3p in GECs by qRT-PCR. Data represent mean±SD (n=3,each). **P<0.01 vs. pre-NC group, ##P<0.05 vs. anti-NC group. D. The effect of cell viability after knockdown RNPC1. Data represent mean±SD (n=3,each). [file 13046_2020_1625_MOESM2_ESM.jpg]
